# Is the immediate effect of marathon running on novice runners’ knee joints sustained within 6 months after the run? A follow-up 3.0 T MRI study

**DOI:** 10.1007/s00256-020-03391-2

**Published:** 2020-02-17

**Authors:** Laura Maria Horga, Johann Henckel, Anastasia Fotiadou, Anna C. Hirschmann, Anna Di Laura, Camilla Torlasco, Andrew D’Silva, Sanjay Sharma, James C. Moon, Alister J. Hart

**Affiliations:** 1 Institute of Orthopaedics and Musculoskeletal Science, University College London and the Royal National Orthopaedic Hospital, Brockley Hill, Stanmore, Middlesex, London, HA7 4LP UK; 2 Department of Radiology and Nuclear Medicine, University Hospital Basel, Basel, Switzerland; 3 Institute of Cardiovascular Science and Barts Heart Centre, University College London, London, UK; 4 Department of Cardiovascular Sciences, St George’s University of London, London, UK

**Keywords:** Marathon running, Knee, MRI, Bone, Cartilage

## Abstract

**Objective:**

To evaluate changes in the knee joints of asymptomatic first-time marathon runners, using 3.0 T MRI, 6 months after finishing marathon training and run.

**Materials and methods:**

Six months after their participation in a baseline study regarding their knee joints, 44 asymptomatic novice marathoners (17 males, 27 females, mean age 46 years old) agreed to participate in a repeat MRI investigation: 37 completed both a standardized 4-month-long training programme and the marathon (marathon runners); and 7 dropped out during training (pre-race dropouts). The participants already underwent bilateral 3.0 T MRIs: 6 months before and 2 weeks after their first marathon, the London Marathon 2017. This study was a follow-up assessment of their knee joints. Each knee structure was assessed using validated scoring/grading systems at all time points.

**Results:**

Two weeks after the marathon, 3 pre-marathon bone marrow lesions and 2 cartilage lesions showed decrease in radiological score on MRI, and the improvement was sustained at the 6-month follow-up. New improvements were observed on MRI at follow-up: 5 pre-existing bone marrow lesions and 3 cartilage lesions that remained unchanged immediately after the marathon reduced in their extent 6 months later.

No further lesions appeared at follow-up, and the 2-week post-marathon lesions showed signs of reversibility: 10 of 18 bone marrow oedema-like signals and 3 of 21 cartilage lesions decreased on MRI.

**Conclusion:**

The knees of novice runners achieved sustained improvement, for at least 6 months post-marathon, in the condition of their bone marrow and articular cartilage.

## Introduction

So far, previous studies have only found few subtle short-term abnormalities, i.e. non-acute lesions, of low grade of severity; on magnetic resonance imaging (MRI) scans of the knees of regular long-distance runners (minutes to few weeks after the marathon); this was where no significant pre-existing injuries were reported in the first place [1–6]. Limited peer-reviewed data on the impact of marathon running over a longer period of time (medium-term, 2–3-month follow-up; long-term, one study 10-year follow-up) has shown that any immediate post-marathon alterations in MRI signal return to baseline in runners within 3 months [5–8]. All follow-up studies up to this point were conducted with a very small population of regular long-distance runners (up to 13 participants; one knee scanned only) [5–9], and none studied the incidence and status of running-related lesions over time in novice runners participating in their first marathon.

To better understand the implications of long-distance running for the knees of novice runners, we aimed to evaluate changes in the knee joints of first-time marathon runners using 3.0 T MRI 6 months after finishing marathon training and run.

## Materials and methods

### Study design and participants

The study received ethical approval by the UK National Health Service (NHS) Research Ethics Committee and informed consent was obtained from all participants. The volunteers were recruited from the group of runners who were successful in the ballot for the Virgin Money London Marathon 2017. Virgin sent emails to all successful marathon entrants and then a call centre was organized to recruit eligible volunteers for the study.

Only those who had participated in the previous study (study 1 [10]) were included in the follow-up investigation (study 2). Forty-four out of the previous cohort of 82 participants returned for study 2. The reasons for dropping out were not linked to their knee condition but to issues of availability to attend the specific MRI scanning days, i.e. the participants were located across the country.

This was a prospective, longitudinal cohort study of 44 healthy asymptomatic volunteers (17 males, 27 females, median age: 45 years), specifically novice runners who signed up for their first marathon. The main inclusion criteria were as follows: volunteers with no previous running experience and physically inactive before the training for their first marathon, i.e. not meeting physical activity requirements of 30 min of moderate-intensity physical activity, 5 days/week, or 20 min of more intense physical activities, 3 days/week, based on existing health recommendations [11–13]; with no present/previous knee injuries or cardiac abnormalities and no contraindications for undergoing MRI. Exclusion criteria included the following: pregnant women, regular long-distance runners, experienced marathon runners, aged < 18 years, with body mass index (BMI) > 30 OR < 18, with known knee problems, previous knee surgeries or poor cardiovascular health. The participants completed The Knee Injury and Osteoarthritis Outcome Score (KOOS) questionnaire [14] to ensure good joint function and no symptoms of knee injury.

All participants took part in a standardized 4-month beginner training plan for the marathon developed by Virgin London Marathon (with gradual increase in mileage, freely accessible online). Out of these 44 participants, 37 completed both the training for/and the marathon run (marathon runners), and 7 dropped-out during training (pre-race dropouts) and did not run the marathon, due to various reasons not linked directly to their pre-training health status: bradycardia (*n* = 1), bronchitis (*n* = 1), calf issue (*n* = 1), and personal (*n* = 4) (see Table 1 for participant characteristics).

**Table 1:** Baseline characteristics of study participants. *BMI*, body mass index; *SD*, standard deviation

Characteristics	Marathon runners *n* = 37	Pre-race dropouts *n* = 7
Age (years)*	46.2 ± 9.3	46.6 ± 4.4
BMI (kg/m^2^)*	24.5 ± 3.4	23.2 ± 1.5
Height (cm)*	169 ± 8.9	177 ± 12.9
Male:female ratio	13: 24	4: 3
Pre-marathon/pre-training low-intensity physical activity (hours/week)^§^	2 (0–4)	2 (0–4)

*Values are reported as mean ± SD for normally distributed data

^§^Mean (range) are reported

### Magnetic resonance imaging

The 44 participants were assessed at all 3 time points: (1) 6 months before the race (and therefore before the training programme; MRI 1), (2) 2 weeks after running the marathon (and post-training; MRI 2), and (3) approximately 6 months later (MRI 3). Both marathon runners and pre-race dropouts were scanned at the same 3 time points. Both knees of all participants were scanned and analyzed independently (88 knee MRI scans). At each time point, measurements were performed with the same 3.0 T MR scanner (Prisma, Siemens Healthcare, Erlangen, Germany) and dedicated 15 channel knee coil for the analysis, and identical parameters of the MRI unit were used in order to achieve optimal comparability. The imaging protocol included proton density–weighted fat suppressed (PD FS) sequences in axial (repetition time msec/echo time in msec; 4630/37), sagittal (4200/41 msec) and coronal planes (5240/41 msec). All slices were 3 mm thick, with an image size/acquisition matrix of 320 × 320 pixels. The total acquisition time per bilateral scan was 25 min and average field of view was 16 cm.

The participants were asked to complete KOOS questionnaires on each MRI scanning day to assess their perceived knee condition including symptoms of functional limitation.

### Radiological reporting

The assessment of all the MRI data was made by a musculoskeletal radiologist with 10-year experience at consultant level (264 MRI scans, 88 knees × 3 time points). The images from half of the cohort (randomly selected participants) were also evaluated independently by a second fellowship-trained musculoskeletal radiologist with 9-year experience at consultant level. Images of each time point were analyzed separately.

Validated scoring/grading systems [15–20] were used to evaluate the MRI findings. Any signal changes/lesions of various grades of severity were quantified for the following knee structures: meniscus [15, 16], cartilage [17], bone marrow [18], tendons [19] and ligaments [16]. All structural subdivisions were assessed. The presence of other findings was specified [20, 21]. All abnormalities were recorded including grade 1 abnormalities (all scores/grades different from 0 will be defined as ‘lesions’ throughout the text). The main three knee compartments (larger units) include the following: patellofemoral joint; lateral tibiofemoral joint; medial tibiofemoral joint. Full details are presented in Table 2.

**Table 2:** Knee scoring/grading systems. *BLOKS*, Boston Leeds Osteoarthritis Score; *ACLOAS*, Anterior Cruciate Ligament OsteoArthritis Score; *KOSS*, Knee Osteoarthritis Scoring System; *MOAKS*, MRI Osteoarthritis Knee Score; *WORMS*, Whole-Organ Magnetic Resonance Imaging Score

Scoring system per knee structure	Scores	
BLOKS (0–7 [15]) and ACLOAS (0–8 [16]): Meniscus (medial, lateral) Anterior horn, posterior horn	BLOKS	ACLOAS
	*Meniscal signal (not a tear)*	0 = Normal meniscus with absence of tear, maceration and hypointense signal
	0 = Absent	
	1 = Present	
	*Type of tear:*	1 = Intrameniscal hyperintensity not extending to meniscal surface
	2 = Vertical tear	
	3 = Horizontal and radial tear	2 = Horizontal tear
	4 = Complex tear	3 = Radial and vertical tear
	5 = Root tear	4 = Bucket-handle tear, displaced tear (including root tears) and complex tears
	6 = Complete maceration	
	7 = Meniscal cyst	
		5 = Meniscal repair
		6 = Partial meniscectomy and partial maceration
		7 = Progressive partial maceration or re-partial meniscectomy (i.e. loss of morphological substance of the meniscus) compared with the previous visit
		8 = Complete maceration or resection
Modified Noyes 0–4 [17]: Cartilage Femur, tibia, patella (medial/lateral) Trochlea (medial, central, lateral)	0 = Normal	
	1 = Grade I lesion: have areas of heterogenous signal intensity on fat saturated IW FSE sequences	
	2 = Grade II lesion: cartilage defects that involve < 1/2 of cartilage thickness	
	3 = Grade III lesion: cartilage defects that involve > 1/2 of cartilage thickness but < full thickness	
	4 = Grade IV lesion: full thickness cartilage defects exposing the bone	
KOSS (0–3 [18]): Bone marrow Femur, Tibia, Patella (medial/lateral) Trochlea (medial, central, lateral)	Bone marrow-oedema like signal	
	0 = Absent	
	1 = Minimal (d < 5 mm)	
	2 = Moderate (d = 5–20 mm)	
	3 = Severe (d ≥ 20 mm)	
Johnson DP et al. (0–3 [19]): Tendons	0 = Normal tendon appearances	
	1 = Increased signal intensity in less than 25% of the axial cross-sectional tendon width	
	2 = Increased high-signal intensity in 25 to 50% of the axial cross-sectional tendon width	
	3 = Increased high-signal intensity occupying more than 50% of the axial cross-sectional tendon width	
ACLOAS 0–3 [16]: Ligaments	ACL and PCL	MCL and LCL
	0 = Normal ligament with hypointense signal and regular thickness and continuity	0 = Continuous ligament with normal signal, no surrounding hyperintensity/oedema
	1 = Thickened ligament and/or high intraligamentous signal with normal course and continuity	
		1 = Continuous ligament with normal signal, surrounding hyperintensity reflecting oedema and/or hematoma
	2 = Thinned or elongated but continuous ligament	
	3 = Absent ligament or complete discontinuity	
		2 = Partial rupture/discontinuity with some preserved fibres
		3 = Complete disruption
WORMS 0–3 [21]: Joint effusion	0 = Absent	
	1 = < 33% of maximum potential distention	
	2 = 33–66% of maximum potential distention	
	3= > 66% of maximum potential distention	
MOAKS 0–3 [20]: Hoffa’s synovitis	0 = Absent	
	1 = Mild	
	2 = Moderate	
	3 = Severe	
MOAKS 0–1 [20]: Other findings	0 = Absent	
	1 = Present	

In case of discrepancies between the radiologists’ reports concerning the findings, agreement (consensus scores) was achieved with a consensus reading in a second MRI reporting session.

### Statistical analysis

Both knees of the same participant were examined and each knee was treated independently in the statistical analysis. Unpaired *t* test was used to assess any significant differences between the two groups (marathon runners versus pre-race dropouts) with regard to age, BMI and height. Chi-square test was used for comparison of gender differences between the two groups, and of differences between the prevalence of lesions in these groups between MRI 1 and MRI 2, and between MRI 2 and MRI 3, respectively. Wilcoxon matched-pairs signed rank test and paired *t* test were used to assess significant differences between the KOOS results recorded at different time points. Statistical significance for analysis was defined as *p* < 0.05 (GraphPad Prism, version 6.0c).

## Results

### Participant characteristics

There were no significant differences between the two groups of volunteers (marathon runners and pre-race dropouts) with regard to age (*p* = 0.922), BMI at the beginning of the study (*p* = 0.238), height (0.060) and gender (0.273).

All marathon runners completed the marathon and the mean finishing time was 5 h 18 min. The physical activity varied among participants in the period of time leading to the 6-month follow-up: marathon runners (mean 3 h/week [0–10]); pre-race dropouts (mean: 2 h/week [0–7]).

No significant differences were found between marathon runners and pre-race dropouts in terms of the prevalence and types of changes between MRI scans, in each of the assessed knee structures (*p* > 0.005). No associations could be made between the participants with sustained lesions at follow-up and other known participant characteristics. There were no significant differences in the participants’ symptoms/perceived knee condition (KOOS scores) over time, throughout the MRI scanning sessions (*p* > 0.05).

### Cartilage

#### Improvement of pre-marathon cartilage lesions

Two pre-marathon cartilage lesions improved in severity grade (2 runners) from MRI 1 to MRI 2: one in the patellofemoral compartment and one in the tibiofemoral one. The improvement was sustained in both cases at MRI 3 (Table 3).

**Table 3:** Prevalence and types of improved pre-marathon lesions at MRI 2, with sustained improvement at MRI 3 (by score/grade of severity), in the cartilage and bone marrow. ‘Improvement’ was defined as reduction in the extent of lesion (score/grade) between MRI scans. The scoring systems were defined in Table 2. *BME*, bone marrow oedema

Knee features per region	Marathon runners				Pre-race dropouts			
	Lesion score/grade			Number of lesions with sustained improvement	Lesion score/grade			Number of lesions with sustained improvement
	MRI 1	MRI 2	MRI 3		MRI 1	MRI 2	MRI 3	
Cartilage lesion								
Patellofemoral	4	3	3	1	–	–	–	–
Medial tibiofemoral	4	2	2	1	–	–	–	–
Lateral tibiofemoral	–	–	–	–	–	–	–	–
Total				2				0
BME-like signal								
Patellofemoral	–	–	–	–	–	–	–	–
Medial tibiofemoral	1	0	0	1	2	0	0	1
Lateral tibiofemoral	2	0	0	1	–	–	–	–
	1	0	0	1	–	–	–	–
Total				3				1

Six months post-marathon, new improvements in the patellofemoral compartment were seen in 2 runners: 3 pre-marathon lesions which were unchanged from MRI 1 to MRI 2 showed improved state at MRI 3. Similarly, in the pre-race dropouts’ group, 3 pre-marathon lesions (in 2 people) improved at MRI 3 (Table 4).

**Table 4:** Prevalence and types of newly improved pre-marathon lesions at MRI 3 (by score/grade of severity), in the cartilage and bone marrow. ‘Improvement’ was defined as reduction in the extent of lesion (score/grade) between MRI scans. The scoring systems were defined in Table 2. *BME*, bone marrow oedema

Knee features per region	Marathon runners				Pre-race dropouts			
	Lesion score/grade			Number of lesions with new improvement at MRI 3	Lesion score/grade			Number of lesions with new improvement at MRI 3
	MRI 1	MRI 2	MRI 3		MRI 1	MRI 2	MRI 3	
Cartilage lesion								
Patellofemoral	4	4	3	2	3	3	1	1
	2	2	1	1	2	2	1	1
	–	–	–	–	1	1	0	1
Medial tibiofemoral	–	–	–	–	–	–	–	–
Lateral tibiofemoral	–	–	–	–	–	–	–	–
Total				3				3
BME-like signal								
Patellofemoral	3	3	2	1	–	–	–	–
	2	2	1	1	–	–	–	–
	2	2	0	1	–	–	–	–
	1	1	0	1	–	–	–	–
Medial tibiofemoral	–	–	–	–	–	–	–	–
Lateral tibiofemoral	2	2	1	1	–	–	–	–
Total				5				0

No further lesions appeared at the 6-month follow-up.

#### Reversibility of post-marathon cartilage lesions

Twenty-one cartilage lesions were found in 13 marathon runners at MRI 2, out of which 13 were new lesions and 8 progressed in extent from the pre-existing lesions at MRI 1; the majority were located in the patellofemoral compartment (17/21; 81%—half were new). Only 4 lesions were observed in the pre-race dropouts’ group (3 participants), mostly in the patellofemoral compartment (3/4; 75%). These lesions were not new but progressed from MRI 1 to MRI 2.

**Fig. 1:**
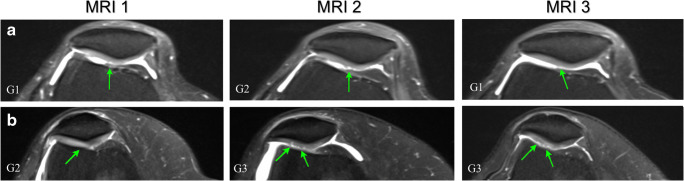
Axial proton-density fat-saturated MR images of two different knees with changes in the extent of chondral lesions of the patella: A) resolution at 6-month follow-up (MRI 3) of a lesion that previously developed from the pre-marathon scan to the 2 weeks post-marathon scan (MRI 1 to MRI 2), in the right knee of a 67-year-old woman; B) smaller lesion at MRI 3 in comparison to MRI 2. The extent of lesion falls within the same grade parameters; however, it is slightly smaller showing signs of reversibility, in the right knee of a 51-year-old woman. Cartilage abnormalities are indicated by arrows and the lesion grade (G) is included in the left bottom corner and is defined in the modified Noyes scoring system [17] (see Table 2)

In the marathon group, 3/21 (14%) cartilage lesions reversed over time, returning to baseline grading status at MRI 3 (Fig. 1; Table 5).

**Table 5:** Prevalence and types of reversible lesions (by score/grade of severity) from MRI 1 through to MRI 2 to MRI 3, in the cartilage and bone marrow. ‘Reversibility’ was defined as resolution/reduction in the extent of those lesions that appeared/progressed at MRI 2 from MRI 1, and then reversed or showed signs of reduction back to MRI 1 grade/score at MRI 3. The scoring systems were defined in Table 2. *BME*, bone marrow oedema

Knee features per region	Marathon runners				Pre-race dropouts			
	Lesion score/grade			Number of lesions that showed reversibility	Lesion score/grade			Number of lesions that showed reversibility
	MRI 1	MRI 2	MRI 3		MRI 1	MRI 2	MRI 3	
Cartilage lesion								
Patellofemoral	0	3	0	1	–	–	–	–
	1	2	1	2	–	–	–	–
Medial tibiofemoral	–	–	–	–	–	–	–	–
Lateral tibiofemoral	–	–	–	–	–	–	–	–
Total				3				–
BME-like signal								
Patellofemoral	0	1	0	2	0	1	0	1
	0	2	0	2	–	–	–	–
	0	3	0	2	–	–	–	–
	0	3	1	1	–	–	–	–
	2	3	1	1	–	–	–	–
Medial tibiofemoral	0	3	2	1	–	–	–	–
Lateral tibiofemoral	0	1	0	1	–	–	–	–
Total				10				1

### Bone marrow

#### Improvement of pre-marathon oedema-like signal

**Fig. 2:**
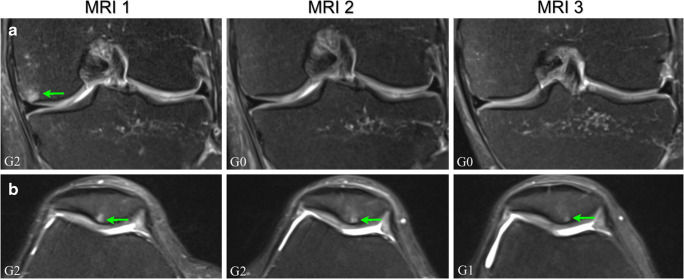
Coronal and axial proton-density fat-saturated MR images of two different knees with changes in the extent of subchondral bone marrow oedema-like signal: A) sustained improvement at 6-month follow-up (MRI 3) of a previous pre-marathon lesion (MRI 1) that reduced in extent 2 weeks after the marathon (MRI 2), in the femur of the left knee of a 54-year-old man; B) new improvement at MRI 3 in a pre-marathon lesion that remained unchanged from MRI 1 to MRI 2, in the patella of the right knee of a 48-year-old woman. Bone marrow oedema-like signal is indicated by arrows and the lesion grade (G) is included in the left bottom corner and is defined in the KOSS scoring system [18] (see Table 2); KOSS, Knee Osteoarthritis Scoring System

Three cases of pre-marathon bone marrow oedema-like signal showed improved condition (reduction in extent) in 2 runners at MRI 2. The improvement was sustained at MRI 3, so it did not reverse back to MRI 1 condition (Fig. 2a). One pre-race dropout also showed improvement of pre-marathon oedema at MRI 2 and this was maintained at MRI 3. All these were seen in the tibiofemoral knee compartment (Table 3).

At MRI 3, new improvements were identified in the patellofemoral compartment in 4 runners: 4 pre-marathon lesions which were maintained from MRI 1 to MRI 2 reduced in extent at MRI 3 (Fig. 2b); and another one that improved was in the tibiofemoral compartment (Table 4).

No further lesions appeared at the 6-month follow-up.

#### Reversibility of post-marathon oedema-like signal

Eighteen bone marrow oedema-like signals were identified in 10 marathon runners at MRI 2: 16 were new and 2 worsened from MRI 1, with the patellofemoral compartment being most affected (15/18; 83%—13 were new lesions). There were 3 new lesions in the pre-race dropouts’ group (2 participants), all in the patellofemoral compartment.

**Fig. 3:**
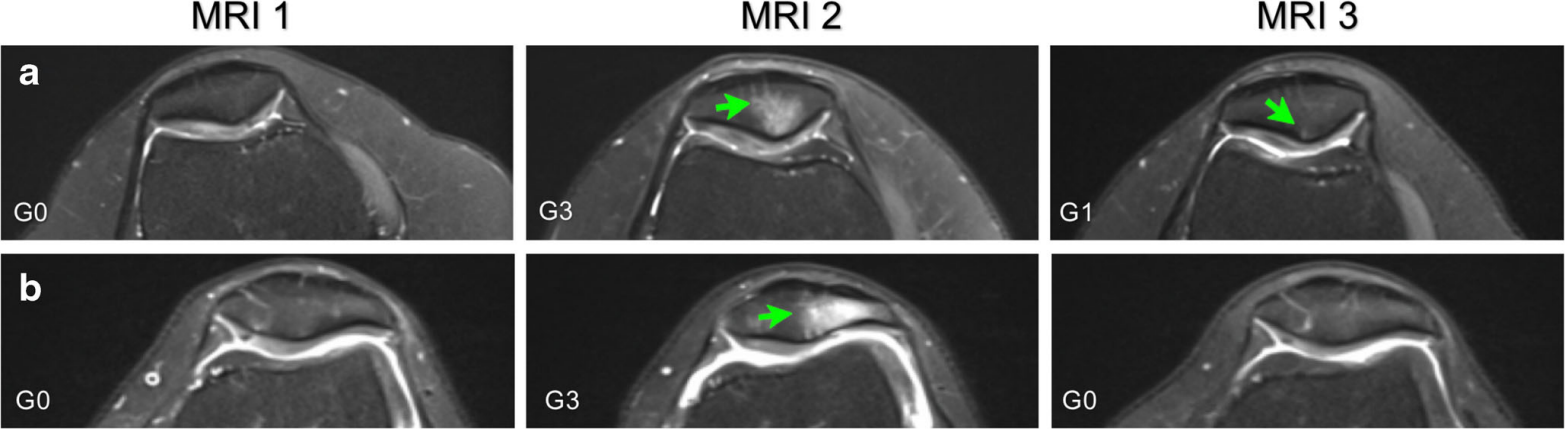
Axial proton-density fat-saturated MR images of two different knees that showed reversibility at 6-month follow-up (MRI 3) in the extent of subchondral bone marrow oedema-like signal of the patella that previously developed from the pre-marathon scan to the 2 weeks post-marathon scan (MRI 1 to MRI 2): A) reversibility but not to the MRI 1 grading status, in the right knee of a 31-year old woman; B) complete resolution to the MRI 1 grading status, in the left knee of a 34-year-old woman. Bone marrow oedema-like signal is indicated by arrows and the lesion grade (G) is included in the left bottom corner and is defined in the KOSS scoring system [18] (see Table 2); KOSS, Knee Osteoarthritis Scoring System

Six months later, 10/18 (56%) bone marrow lesions showed reversibility over time, with 8 of them returning to the pre-marathon state (Fig. 3; Table 5). In the pre-race dropouts’ group, 1/3 (33%) lesions discovered at MRI 2 showed reversibility.

### Other findings

Four cases of semimembranosus tendon signal hyperintensity were seen at MRI 2 and one of them showed reversibility at MRI 3. Two ligamentous lesions were discovered in 2 marathon runners at MRI 2 and both reversed at MRI 3. No further development of other lesions was observed.

## Discussion

Our study demonstrated that both the training for/and the marathon run may be linked with sustained improvement/regression of pre-marathon bone marrow oedema-like signal and cartilage lesions in novice runners within 6 months after the run. No further lesion acquisition was observed at follow-up, and the few immediate post-marathon lesions showed signs of reversibility. There were no significant differences between marathon runners and pre-race dropouts in terms of results, suggesting that MRI changes may not be attributed to the marathon run alone but to the training as well. This is the first study to show sustained beneficial effect of marathon running on MRI at a 6-month follow-up.

The data adds to the existing literature for the following reasons: (1) the study is the largest to date to assess the effect of marathon running over time using 3.0 T MRI, with the longest medium-term follow-up. Previous MRI studies involved ≤ 13 runners, follow-ups of up to 3 months and none suggested permanent running-related damage or any sustained beneficial effect on knees [5–8]; (2) our cohort included first-time marathoners, with no running experience before the marathon training, whereas the runners in previous studies had long-distance running experience; (3) the impact of both the training for and the marathon run was assessed, while most previous work studied the knee joints shortly before and after the marathon day, not before training.

We acknowledge the following study limitations: (1) the activity levels of all participants at the beginning of the study and at follow-up were self-reported. The participants could have varied their activity levels and this might have affected the recovery of some lesions more than others; (2) non-runner controls were not involved; however, we included the dropouts from training who did not run the marathon in our analysis; (3) the exact times of dropping out from training by pre-race dropouts were unavailable and could not be commented on; (3) longer term follow-up studies are still needed to clarify the fate of improved lesions in relation to participant characteristics over time, as well as whether complete resolution of remaining lesions occurs later on.

The sustained beneficial effect of running on knees at 6 months after the marathon implies that running may help in reducing the chances of osteoarthritis in the long term. Few other (non-MRI) studies suggested running may protect the knee joint from osteoarthritis [22–25]. Any remaining bone marrow oedema-like signal appearing post-marathon is expected to resolve within 2 years [16, 26–30]. The cartilage may be able to adapt to loads caused by repeated loading during running but recovery time may vary [3, 31].

In conclusion, the knees of first-time marathoners achieved sustained improvement, for at least 6 months post-marathon, in the condition of the bone marrow and articular cartilage.
